# One-year follow-up of a randomized trial with a dilemma-focused intervention for depression: Exploring an alternative to problem-oriented strategies

**DOI:** 10.1371/journal.pone.0208245

**Published:** 2018-12-13

**Authors:** Guillem Feixas, Clara Paz, Eugeni García-Grau, Adrián Montesano, Joan C. Medina, Arturo Bados, Adriana Trujillo, Eliana Ortíz, Victoria Compañ, Marta Salla, Mari Aguilera, Víctor Guasch, Jordi Codina, David A. Winter

**Affiliations:** 1 Department of Clinical Psychology and Psychobiology, University of Barcelona, Barcelona, Spain; 2 The Institute of Neurosciences, University of Barcelona, Barcelona, Spain; 3 School of Psychology, Universidad de Las Américas, Quito, Ecuador; 4 Department of Psychology and Educational Sciences, Open University of Catalonia, Barcelona, Spain; 5 Faculty of Psychology, El Bosque University, Bogotá, Colombia; 6 Faculty of Psychology, Universidad Católica de Colombia, Bogotá, Colombia; 7 Mental Health Services, Fundació Sanitària Sant Pere Claver, Barcelona, Spain; 8 Department of Psychology and Sport Sciences, University of Hertfordshire, Hatfield, United Kingdom; TNO, NETHERLANDS

## Abstract

**Background:**

Cognitive behavioural therapy (CBT) is aimed to counteract cognitions and behaviours that are considered as dysfunctional. The aim of the study is to test whether the inclusion of a non-counteractive approach (dilemma-focused intervention, DFI) in combination with CBT group therapy will yield better short- and long-term outcomes than an intervention conducted entirely using CBT.

**Method:**

A total of 128 patients with depression and at least one cognitive conflict, of six health community centres in Barcelona, participated from November of 2011 to December of 2014 in seven weekly group CBT sessions and were then randomly allocated to either DFI or CBT (eight individual sessions each) by an independent researcher. Depressive symptoms were assessed with the Beck Depression Inventory-II at baseline, at the end of therapy and three- and twelve-month follow-ups. Therapists did not participate in any of the assessments nor in the randomisation of patients and evaluators were masked to group assignment. Both intention to treat and complete case analyses were performed using linear mixed models with random effects.

**Findings:**

According to intention-to-treat analysis (*F*_*2*, *179*_ = 0.69) and complete case analysis *(F*_*2*, *146*_ *=* 0.88), both conditions similarly reduced the severity of symptoms across posttreatment assessments. For the 77 participants (CBT_group_ +CBT_individual_ = 40; CBT_group_+DFI_individual_ = 37) that completed allocated treatment and one-year follow-up assessment, response and remission rates were relative higher for the DFI condition, however no significant differences were found between treatment conditions. The relapse rates were similar between treatment conditions (CBT_group_ +CBT_individual_ = 7/20; CBT_group_+DFI_individual_ = 8/22).

**Interpretation:**

Although using a counteractive approach across all the treatment sessions is quite effective, it does not seem to be necessary to produce significant improvement. DFI may be considered as an alternative, which could be included in a wider treatment for depression.

**Trial registration:**

ClinicalTrials.gov; ID: NCT01542957.

## Introduction

Cognitive behavioural therapy (CBT) has become one of the most studied and most widely practiced psychological interventions. Specifically, for depression, it is recommended in clinical guidelines [1] as the first choice psychological intervention for adults. Moreover, several studies suggest that cognitive behavioural therapy has enduring effects which reduce recurrences and relapses after treatment termination [2].

One of the basic assumptions of the CBT model [3] is that thinking and behaviours play a significant role in the etiology and/or maintenance of psychological disorders. The aim of the treatment is to modify dysfunctional cognitions and behaviours to promote more adaptive responses, often using problem-solving. Particularly, for depression, the target is to challenge negative thinking and behaviours using a variety of techniques aimed to counteract them. Although modification/correction of cognitive distortions (e.g., “I cannot do anything right”) is considered as a necessary mediator of symptom improvement, little empirical evidence supports this claim [4]. Indeed, Wampold [5], reviewing the evidence, has gone so far as to say that ‘the ingredients of the most conspicuous treatment on the landscape, cognitive-behavioural treatment, are apparently not responsible for the benefits of this treatment’ (pp147-148). It may be, therefore, that other interventions, not necessarily counteractive in nature, might also result in positive therapeutic outcomes.

One method which is not based in counteracting negative thinking and behaviours is dilemma-focused intervention (DFI) [6]. Rather than aiming to correct logical errors of thinking, DFI understands them as necessary constructions of meaning which allow the individual to maintain a sense of continuity, essential for their identity. Instead of counteracting distorted cognitions, doing problem-solving, or promoting pleasurable activities, DFI aims to explore personal meanings and how they can represent a dilemma, or cognitive conflict, between the desire for change and that for continuity. Thus, the objective of this approach is to help patients to become aware of such dilemmas and assist them in finding a resolution [7], which is satisfactory for them.

**Fig 1:**
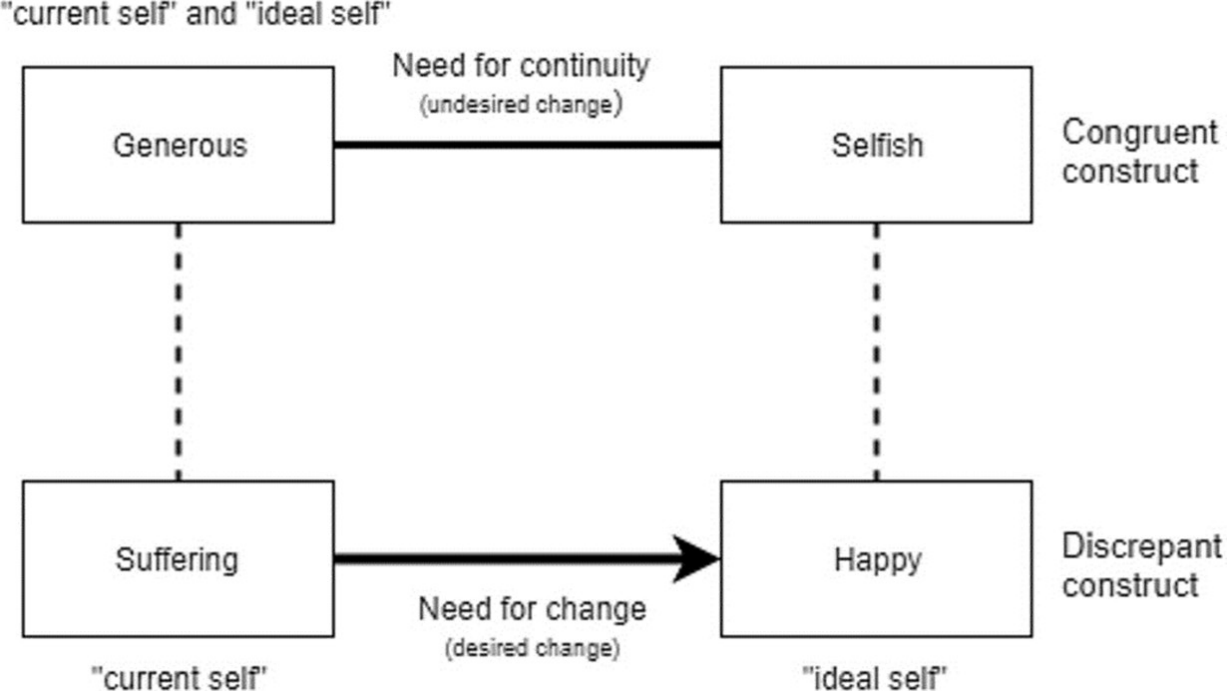
Example of an implicative dilemma found in the repertory grid of one of the participants of the study.

DFI uses the repertory grid technique (RGT), which has a long tradition within personal construct theory [8] as a way to identify dilemmas [9]. This instrument permits the identification of personal constructs, bipolar dimensions (e.g., “anxious” versus “calm”*)* which the individual applies to the self and significant others as well as to their ideal self, and exploration of the relationships between these constructs. A dilemma emerges when a desired change (movement of the self towards the ideal self) on one construct, here termed as *discrepant construct*, is related to an undesired change (movement of the self away from the ideal self) on another construct, a *congruent construct*. In such cases, personal change such as the loss of a symptom is hindered because it threatens personal continuity and thus identity. For example, in the case illustrated in Fig 1, the person sees their current self as “suffering” but their ideal self as “happy” (discrepant construct) but loss of suffering poses the dilemma that, although their current and ideal selves are both seen as “generous” rather than “selfish” (congruent construct), the relationship between these two constructs for the individual is such that “happy” people are viewed as “selfish”. If the person were no longer to suffer, they would therefore risk the undesired change of having to see the self as “selfish”. Previous studies have indicated remarkable differences between depressed patients and controls regarding both prevalence and number of dilemmas [10, 11]. The work of the first sessions of this intervention is to formulate the presenting problem in relation to the dilemma(s), often implicit, detected with the RGT. The following sessions involve the use of a variety of techniques [6, 12] aimed at assisting patients to explore and resolve their dilemmas. By focusing therapy on the dilemmas instead of pushing patients towards a direct change regarding dysfunctional behaviours and thinking patterns, DFI promotes the exploration of the implications of the desired change. This exploration is made in a way that reveals the person’s need for continuity (not only their need for change) in his or her sense of identity, despite the suffering and malfunction that keeping that coherence or continuity may involve.

Intervention is understood as a delicate renegotiation of the personal meanings of the patient and of the positive and negative implications of the symptom or problem as they configure the dilemma, without opting for one a priori solution. The therapist adopts a facilitating role in this exploration so the person can rebuild his or her life solving their own dilemmas. Ultimately, patient and therapist should jointly generate constructions appropriate to the unique and personal demands of each patient. Therefore, the interventions do not seek to attain a stereotyped result or previously defined outcome, rather to make compatible the patient’s desired change with the maintenance of his or her sense of identity. It is not intended to unfailingly change the symptomatic construct, but rather to resolve the personal dilemma in the terms the patient considers more suitable for him or her.([6], p 28).

The principal objective of the current study was to assess the long-term benefits (one year after termination of the treatment) of two types of treatment conditions for depression that combine group and individual modalities. One of these conditions relied almost exclusively on a counteractive approach towards symptoms (CBT_group_ + CBT_individual_), while the other condition combined a counteractive approach in the group part of the treatment (CBT) with a non-counteractive approach in the individual part, which focused on the resolution of internal conflicts or dilemmas (DFI). Thus, in both conditions patients participate initially in group CBT and are, then, randomly assigned to individual sessions of either CBT or DFI. Results at post-treatment and three-month follow-up [13] showed a significant reduction in symptoms and psychological distress for both conditions. However, there were no significant differences between them. In this study, we compare follow-up outcomes one year after the treatment regarding severity of symptoms and occurrence of relapses.

If both conditions again attain equivalent effects in terms of both depressive symptom reduction and relapse and recovery rates, the results would invalidate claims of superiority of either of the two approaches but also indicate that different, even in some respects opposing, change strategies can be adopted for a successful psychotherapeutic treatment of depression.

## Materials and methods

### Study design

This study followed a controlled trial methodology to evaluate the efficacy of treatments [14]. This trial was conducted in Barcelona, at several primary and mental health centres (CSMA Nou Barris Nord, Associació Catalana de Teràpies Cognitives, Hospital de Mataró, and others belonging to Parc de Salut Mar and Fundació Sant Pere Claver). In total, 22 groups were formed with between four and nine patients in each group [13].

This study was approved by the Committee for Ethics in Research of the University of Barcelona under the number (IRB00003099) on July 2011 and also by the ethical committees of the other centres involved in the study (for detailed information about study protocol see https://www.ncbi.nlm.nih.gov/pmc/articles/PMC3659066/). This study was registered at ClinicalTrials.gov under number NCT01542957 and also at Current Controlled Trials (ISRCTN92443999) [14] on February, 2012. Recruitment of participants started on November 2011 after the approval of the Committee for Ethics, but registration of the trial was delayed until permissions and arrangements of all the centres involved were ready. The authors confirm that all ongoing and related trials for this intervention are registered.

### Participants

Patients were recruited by advertisements and by referrals from the above-mentioned centres from November 7^th^, 2011, to December 19^th^, 2014. Inclusion criteria were (1) age range between 18 and 70 years old; (2) score of 20 or above on the BDI-II; (3) meeting diagnostic criteria for major depressive disorder or dysthymia according to the Diagnostic and Statistical Manual of Mental Disorders, 4th edition, text revision (DSM-IV-TR), [15] and having at least one cognitive conflict detected by the application of the RGT. Exclusion criteria were (1) psychotic symptoms, manic or hypomanic episodes in the past, substance abuse, organic brain dysfunction, acute suicidal ideation, and mental retardation; (2) already receiving psychological treatment; (3) substantial visual, hearing, and cognitive deficits; and (4) insufficient linguistic competence to communicate in Spanish or Catalan [13].

At the initial assessment session, the nature of the study was explained and, if the patient agrees to participate, he signed the informed consent, and then the BDI-II was applied to verify the clinically significant presence of depressive symptoms (a score of 20 or above) [14].

All of the 106 patients who completed the allocated treatment (mean age 49.9 years, SD = 11.09; 79.2% females) were contacted one year after the completion of the trial and 77 (72.6%) agreed to be assessed for this study.

### Randomisation and masking

Randomisation to the eight individual sessions using either a CBT or a DFI manual was carried out after the completion of the CBT group phase, and so therapists had no knowledge of patient allocation until they called them for individual therapy. Random assignment of the patients who had completed this group phase was done using permuted blocks. Each block consisted of the participants of a particular centre which had enough patients to initiate a group (usually 6) with a 1:1 allocation ratio. A staff member of the Department of Clinical Psychology and Psychobiology of the University of Barcelona, completely oblivious to the study and blind to treatment conditions, assigned patients either to treatment “A” or “B” and, then, the researcher in charge revealed to the therapists of each condition (CBT for those in “A”, DFT for those in “B”) the corresponding names of the patients. In any case, therapists did not participate in any of the assessments nor in the randomisation of patients, and evaluators were blind to group assignment [14].

### Procedures

Treatment conditions are described in more detail in the initial report of this trial [13]. All participants started the treatment receiving seven two-hour sessions of group CBT for depression. Once they concluded this part of the treatment, they were randomly allocated to receive eight sessions of individual therapy using either CBT or DFI. The treatment ended with one CBT two-hour group session, this making a total of 16 sessions. The two types of individual therapy were tested for therapist adherence to the respective manuals with satisfactory results.

Cognitive behavioural therapy was used in the group part of the treatment and the individual part for one of the conditions. Specific manuals (available from the authors upon request) were created for the current study based on Beck’s cognitive therapy manual [16] and other CBT materials. Techniques employed included cognitive restructuring, activity scheduling, behavioural experiments, imagery modification, and relapse prevention. For DFI, a specific manual was designed for this study [6, 12]. This individual intervention is based on personal construct theory [17] and its objective is to achieve dilemma resolution mainly by promoting reconstruction of personal meanings about self and others. Techniques used to achieve this included self-characterization, identification of dilemma’s prototypical figures, laddering, magic wand, reconstruction of immediate experiencing as a function of the dilemma, analysis of the relational implications of the dilemma, historical reconstruction of the dilemma, dramatic representation of the dilemma (two-chair dialogue), and future projection (life without the dilemma). It was applied in one of the arms of the study. In both approaches, much attention was paid to the establishment and quality of the therapeutic relationship.

### Outcomes

At the one-year follow-up assessment two psychologists administered the structured clinical interview for DSM-IV axis I (SCID-CV), [18] which was also used at baseline and at posttreatment for diagnostic purposes. Absence of recovery was considered to be indicated when participants met criteria at both assessment points. Relapse was considered to be indicated when participants did not meet criteria after the treatment but did so at one-year follow-up. Maintenance was considered to be indicated when patients did not meet criteria either after therapy or at follow-up. Improvement was considered to be indicated for participants who were not recovered after therapy but did not meet criteria for major depression or dysthymia at follow-up.

The 21-item Beck Depression Inventory (BDI-II) [19, 20] was applied to assess the severity of depression at every assessment point (baseline, posttreatment, three-month and one-year follow-ups). This measure was used to determine response (decrease from pre- to follow-up assessment of ≥ 47%) and remission rates at one year follow-up (BDI-II total score ≤ 12) [21].As described by Feixas and Saúl [9], repertory grid technique (RGT), with an associated software package [22], was used to identify the presence of dilemmas, information which was used by therapists applying DFI. Essentially, the technique involves eliciting the individual’s personal constructs by asking them to compare and contrast the self, ideal self and significant others (e.g., parents, siblings, partners, friends), who are then rated on a 7-point scale on each of the constructs elicited. It is important to note that the dimensions on which the interviewee rates the self and others are not pre-established items (as in traditional questionnaires) but rather their own words (personal constructs) elicited in the conversation which arises when asked for similarities and differences among the self and significant others in their lives.

### Statistical analyses

To determine the sample size, we calculated that, with a statistical power of 0.80, considering a dropout rate of 20%, an α significance level of 0.05 (two-sided) and an effect size of 0.30, we require a total of 112 patients. In terms of our primary outcome measure (BDI-II), and based on Spanish normative data, calculation yielded a 3.3-point difference between groups [14].

The primary outcome measure (BDI-II) was analysed using random effects mixed models for both intention-to-treat (ITT) participants (*N* = 128) and for complete cases (*n* = 77; those who completed the treatment and also the one-year follow-up assessment). The models considered maximum likelihood estimation and autoregressive covariance structure, using unbiased estimates when having missing data assuming that data was missing randomly. Symptom reduction after treatment and at follow-ups was compared between both conditions (CBT_group_ + CBT_individual_ vs. CBT_group_ + DFI_individual_). The model specified treatment (CBT_group_ + CBT_individual_ vs. CBT_group_ + DFI_individual_), time (post-treatment, 3-month follow-up and one-year follow-up), and the interaction between type of treatment and time as fixed effects, while controlling by pre-treatment scores. Participants were considered as random effects. Pairwise comparisons were used to test differences between treatment conditions in each assessment point. Test significance was set at *p* < 0.05 (two-tailed).

As secondary outcome, the remission, response and relapse rates were analysed. Relative effect (relative risk) and the absolute effect (risk differences) with 95% confidence intervals were calculated for each treatment condition at each posttreatment assessment point. Data analyses were performed using the version 3.5.1 of “R” statistical software [23].

## Results

**Fig 2:**
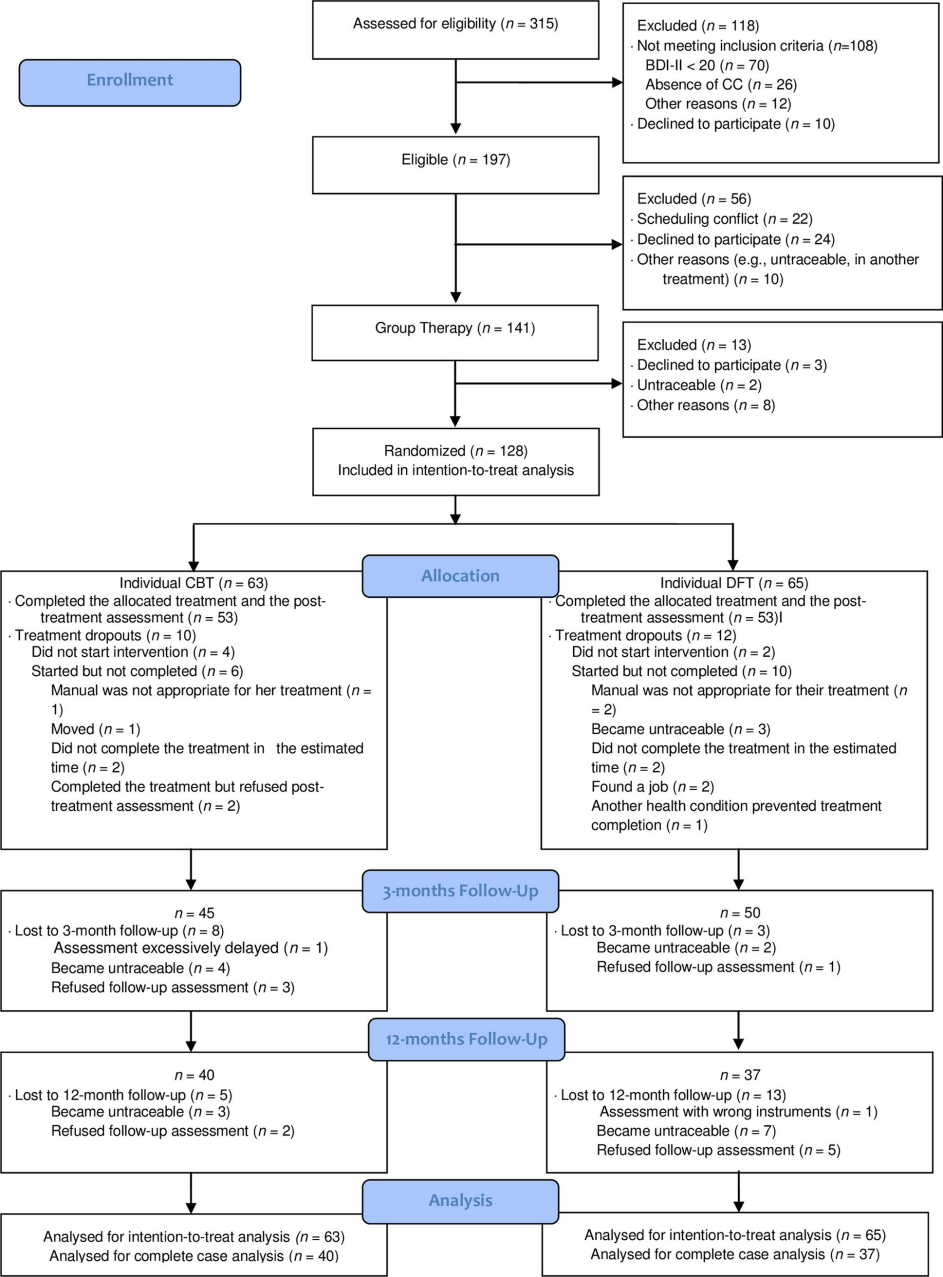
CONSORT diagram of participants including one-year follow-up assessment. BDI-II = Beck Depression Inventory-II; CC = Cognitive Conflict; CBT = Cognitive Behavioral Therapy; DFI = Dilemma-Focused Intervention.

This study was conducted from the 7^th^ of November, 2011, to the 19^th^ of December, 2014. Seventy seven participants (72.64% of those who completed treatment; CBT_group_ + CBT_individual_ = 40, CBT_group_ + DFI_individual_ = 37) were assessed at one-year follow-up. The percentage of participants assessed at one-year follow-up was evenly distributed across conditions (CBT_group_ + CBT_individual_ = 75.5%, CBT_group_ + DFI_individual_ = 69.8%; χ^2^ (1,*n* = 106) = 0.42). Complete cases had a mean age of 49.06 years (*SD* = 11.61), and 77.9% (60/77) were females. Table 1 displays baseline demographic characteristics for completers and intention-to-treat participants according each allocated treatment. The baseline demographic characteristics did not differ between treatment conditions. Moreover, when comparing those who completed the one-year follow-up and those lost in that assessment, no significant difference was found for the baseline characteristics, the only exception was the civil status (χ^2^ (1,*n* = 106) = 5.53), for which more participants married/cohabiting completed the one-year follow-up assessment (*n* = 48, 69.6%). The design of the clinical trial is depicted in Fig 2.

**Table 1:** Baseline, demographic and clinical characteristics of participants.

	Intention to treat				Completers			
	CBT_group_ + CBT_individual_ (n = 63)		CBT_group_ + DFI_individual_ (n = 65)		CBT_group_ + CBT_individual_ (n = 40)		CBT_group_ + DFI_individual_ (n = 37)	
Characteristic	*M (SD)*	*n(%)*	*M (SD)*	*n(%)*	*M (SD)*	*n(%)*	*M (SD)*	*n(%)*
Age (in years)	50.06(11.03)		48.37(11.22)		50.10(11.37)		47.95(11.91)	
Gender								
Female		48(76.2)		51(78.5)		31(77.5)		29(78.4)
Male		15(23.8)		14(21.5)		9(22.5)		8(21.6)
Marital status								
Single/Widowed/Divorced		32(50.8)		27(41.5)		24(60.0)		24(64.9)
Married/Cohabiting		31(49.2)		38(58.5)		16(40.0)		13(35.1)
Education								
Elementary (6 years)		14(22.2)		9(14.28)		10(25.0)		4(10.8)
High school (7–12 years)		23(36.5)		19(29.2)		15(37.5)		10(27.0)
More than 12 years		26(41.3)		37(56.9)		15(37.5)		23(62.2)
Work status								
Active		18(28.6)		20(30.8)		10(25.0)		10(27.0)
Inactive		45(71.4)		45(69.2)		30(75.0)		27(73.0)
BDI-II	37.05(10.07)		36.31(9.16)		37.35(9.32)		36.08(8.71)	
GAF	56.68(7.10)		57.77(7.35)		56.68(7.10)		57.77(7.35)	
Diagnosis								
MDD		31(49.2)		27(41.5)		22(55.0)		17(45.9)
MDD-R		29(49.2)		32(49.2)		16(40.0)		16(43.2)
Dysthymia		3(4.8)		6(9.2)		2(5.0)		4(10.8)
Previous suicide attempts								
Yes		15(23.8)		21(33.3)		9(22.5)		6(16.2)
No		48(76.2)		42(66.7)		31(77.5)		31(83.8)
Psychiatric medication								
Yes		48(76.2)		51(78.5)		29(72.5)		25(69.4)
No		15(23.8)		14(22.7)		11(27.5)		11(30.6)
Chronicity^a^	11.50(11.58)		11.33(11.76)		11.59(12.05)			11.22(11.85)

^a^ Chronicity = number of years from the first depressive episode

CBT = Cognitive Behavioral Therapy; DFT = Dilemma-Focused Therapy; CI = Confidence Interval.

### Treatment efficacy measured with the BDI-II

Following the ITT principle, all randomized participants (*N* = 128) were analysed taking their BDI-II scores at posttreatment and two (3-month and 1-year) follow-up time points. The results showed no statistically significant change of severity of depression over time at posttreatment and follow-up assessments when controlling for baseline scores, *(F*_*2*, *179*_ = 0.69). In addition, no significant effects were found between treatments, when the type of treatment (*F*_*1*, *110*_ *=*_*-*_0.04) and the interaction between time and type of treatment condition was entered in the model (*F*_*2*, *179*_ *=*_*-*_0.78). Pairwise comparison revealed no significant effects of treatment conditions at post-treatment, three-month and one-year follow-up.

Similar results were obtained by considering only the data of those who completed the treatment and one-year follow-up assessment (*n* = 77). Symptoms did not significantly change at posttreatment and follow-up assessments when controlling for baseline scores (*F*_*2*, *146*_ *=* 0.88). Also, no significant effects were identified between treatment conditions (*F*_*1*, *74*_ = 0.09) and the time by group interaction (*F*_*2*, *146*_ *=* 0.68). As with the ITT analysis, the pairwise comparison indicated that there were not significant effects between treatment conditions at each assessment point. Therefore, there is no support for claiming a differential effect between conditions. Means and standard deviations are presented in Table 2 for each treatment condition for ITT and completers; furthermore, the estimated mean difference and their 95% confidence intervals are included for between subjects’ comparisons at each assessment point after the treatment while controlling for baseline scores.

**Table 2:** Outcome data at each posttreatment assessment point and results from pairwise comparisons between treatment conditions and each assessment point corrected for baseline values.

	Treatment condition				
Time	CBT_group_ + CBT_individual_	CBT_group_ + DFI_individual_	Comparison between treatment conditions		
	BDI-II Mean (*SD*)	BDI-II Mean (*SD*)	Mean Estimate of adjusted effects between treatment conditions ^a^	*p*-value	95% CI
Intention–to-treat sample (*N* = 128)					
Post-treatment	23.71(15.22)	21.60(15.50)	1.25	0.61	-3.72, 6.23
Three-month follow-up	23.88(15.95)	23.00(15.04)	-1.39	0.59	-6.63, 3.84
One-year follow-up	22.58(15.67)	21.03(15.01)	1.45	0.60	-4.08, 6.98
Complete cases (1-year follow-up: *n* = 77)					
Post-treatment	21.41(14.65)	19.60(15.49)	1.70	0.56	-4.19, 7.60
Three-month follow-up	21.34 (15.53)	21.80(15.61)	-1.17	0.69	-7.16, 4.82
One-year follow-up	20.34 (14.19)	17.71(14.24)	1.77	0.55	-4.13, 7.67

Note. BDI-II = Beck Depression Inventory-II; CI = Confidence Interval; CBT = Cognitive Behavioural Therapy; DFI = Dilemma-Focused Intervention.

^a^ Values are adjusted

In addition to this analysis, the effect of medication on outcome was explored for the whole sample. At intake, 36 participants (28.1%) reported not taking antidepressant medication but they reduced their symptoms similarly to those who were taking medication (*F*_*1*, *140*_ = 0.01). Also here, no differential effects were found between treatment conditions, (*F*_*2*, *139*_ = -0.25). At one-year follow-up, 66 participants informed their medication status from the beginning of the treatment. Nineteen participants indicated not having taken antidepressants during the treatment, while 47 declared to have taken them. No differential effects were found in the reduction of depressive symptoms between participants who took medication and those who did not, (*F*_*1*,*122*_ = -0.33). Also, there were no significant differences between treatment conditions (*F*_*1*, *120*_ = 1.03).

### Response and remission

Additional to the primary outcome the response and remission rate was calculated for each assessment point (post-treatment, three-month follow-up and one-year follow-up) by each treatment condition. Table 3 shows the number of participants, percentage, risk ratio (relative effect) and risk difference (absolute effect) between treatment conditions. The results indicated that the inclusion of individual DFI increased the chance of response in the three assessment points compared with the treatment that only included CBT. However, the difference in response rate between treatment conditions was not significant in any assessment point after treatment. Similarly, the chance of remission was higher when including individual DFI in the treatment, but there were not significant differences between treatment conditions in any assessment point.

**Table 3:** Response and remission rates at each assessment point after randomization by treatment condition.

	Response				Remission			
	Percentaje (*n*)		Risk		Percentaje *(n*)		Risk	
Assesment point	CBT_group_ + CBT_individual_	CBT_group_+ DFI_individual_	Ratio (95% CI)	Difference (95% CI)	CBT_group_ + CBT_individual_	CBT_group_+ DFI_individual_	Ratio (95% CI)	Difference (95% CI)
Post-treatment	n = 53	n = 53			n = 53	n = 53		
	43.4 (23)	58.5 (31)	1.34(0.91, 1.97)	-15.1(-32.56, 3.81)	34.0 (18)	39.6(21)	1.16(0.70, 1.92)	-5.6(-23.20, 12.38)
Three-month follow-up	n = 45	n = 50			n = 45	n = 50		
	48.8 (22)	50.0 (25)	1.02(0.68, 1.53)	0.00(-20.41, 18.31)	31.1 (14)	32.0 (16)	1.02(0.56, 1.86)	-0.9(-18.91, 17.50)
One year follow-up	n = 40	n = 37			n = 40	n = 37		
	47.5 (19)	59.5 (22)	1.25(0.82, 1.91)	-11.96(-32.30, 9.95)	37.5(15)	45.9(17)	1.22(-1.08, 027)	-8.45(-28.98, 13.04)

Note. CI = Confidence Interval.

### Relapses

Of the 77 participants who completed the assessment at one-year follow-up, 42 (*n*_*CBTgroup + CBTindividual*_ = 20, *n*_*CBTgroup + DFIindividual*_ = 22) did not meet criteria for major depression or dysthymia at the end of the intervention when they were assessed using SCID-CV [18]. However, 15 (35.7%) showed relapse at one-year follow-up (*n*_*CBTgroup + CBTindividual*_ = 7, *n*_*CBTgroup + DFIindividual*_ = 8). The relative risk of having a relapse was greater by a factor of 1.03 (95% CI = 0.46, 2.34) in the condition that included DFI, however the risk difference between treatment conditions was negligible (Risk Difference = -0.01, 95% CI -0.28, 0.26).

## Discussion

Results of the present study revealed no significant differences between treatment conditions with respect to depressive symptom reduction. Furthermore, the relapse rate was similar for both conditions. Thus, the inclusion of individual sessions of a non-counteractive module (DFI) combined with a counteractive treatment (CBT) was not, as initially predicted, superior to the use of counteractive strategies across all the treatment. Therefore, we can regard both counteractive techniques (CBT) and those which focus on dilemmas (and avoid prescribing positive thoughts and behaviours) as equivalent in their efficacy. It is also noteworthy that there was no evidence that taking anti-depressant medication influenced treatment outcome.

This is the first study evaluating the efficacy of a combined treatment including group CBT and individual DFI. For this purpose, an active (and prestigious) control condition was used, treatment manuals were developed and followed, therapists’ adherence checks performed, evaluators were blind and independent from therapists, and the study included a reasonably long follow-up assessment. In favour of the external validity of the study, we must notice that inclusion criteria were not very restrictive and patients were drawn from and seen in their usual health care centres. Finally, to control for investigator’s allegiance and to promote high treatment quality in both arms the research team involved in the study included senior members with extensive experience in each of the treatment conditions being tested.

Some limitations need to be noted for this study. The design of the interventions is set up in a way in which half of the sessions (all the group ones) are held in common and, thus, the differential interventions (CBT or DFI) only include the other half (eight individual sessions). This might have limited the chances to obtain differential effects. Future studies having a substantial part of the treatment in common for patients in the two arms might do well to supplement the sample size that would be estimated for ordinary clinical trials comparing two completely different approaches from session one to the end of therapy. The fact that a self-report measure was used as the primary outcome can be regarded as a limitation of the study.

Although patients were not informed which type of individual therapy they had been assigned to, we cannot ensure that they were absolutely blind to the treatment condition. However, they had little information to discriminate between the two treatment modalities of our study (both individual and with a strong cognitive emphasis) and, thus, we can assume that should there have been some influence derived from patient awareness of the assigned therapy, it must have been very small. Another limitation is that contact of patients with other treatment options during the follow-up period was not controlled.

A characteristic of this study which should be taken into account is that only 19 participants (15% of those who were randomised) were under 30 years of age. Of those, only six (32%) completed the treatment and follow-up measures, which suggests a serious engagement problem. These facts indicate young adults were misrepresented in our study. Further studies would do well in targeting young adults and promoting strategies for increasing treatment adherence, a factor which is widely recognised as essential to attain good therapeutic outcomes.

These limitations, however, do not prevent consideration of these results as evidence that the consistent application of counteractive interventions or techniques in psychotherapy for depression (as in CBT) is not a necessary ingredient to generate significant changes with respect to symptom severity and relapse rates. Therefore, non-counteractive approaches such as DFI may be regarded as valid interventions for the treatment of depression, for instance if a patient’s responses or attitudes during the therapy process suggest that personal dilemmas may be blocking change, or even when the therapist feels more comfortable using these alternative techniques or attitudes. Actually, several studies [24–26] suggest that therapists choose therapeutic approaches which match their philosophical beliefs and ‘personal styles’, and it might be that they were more effective when using such approaches than when using approaches which do not match their beliefs and styles. Conceivably, therefore, some therapists are more likely to achieve good outcomes when using a counteractive, and others when using a non-counteractive, approach.

Further studies could research whether non-counteractive strategies such as DFI could be efficacious by themselves (without including counteractive or problem-solving strategies). Also, we can anticipate that for some patients a non-counteractive approach would be more facilitative for promoting self-generated change while others would benefit more from direct prescriptions or directions. In fact, this idea has already received some empirical support [24–27], and new research might usefully be directed at developing assessment tools to tap patient preferences for counteractive or non-counteractive approaches, which could be used when designing a treatment plan for each patient. In any case, it seems desirable that mental health providers offer both counteractive and non-counteractive evidence-based psychotherapy interventions for patients to match their preferences and also the therapists’ styles [28].

## Supporting information

S1 CONSORT Checklist (DOC)

S1 Study Protocol (PDF)
